# The Potential of Functional Hydrogels in Burns Treatment

**DOI:** 10.3390/gels11080595

**Published:** 2025-07-31

**Authors:** Nathalie S. Ringrose, Ricardo W. J. Balk, Susan Gibbs, Paul P. M. van Zuijlen, H. Ibrahim Korkmaz

**Affiliations:** 1 Department of Plastic Reconstructive and Hand Surgery, Amsterdam UMC, Location VUmc, 1081 HZ Amsterdam, The Netherlands; n.s.ringrose@student.vu.nl (N.S.R.); r.w.j.balk@amsterdamumc.nl (R.W.J.B.); p.vanzuijlen@amsterdamumc.nl (P.P.M.v.Z.); 2 Department of Molecular Cell Biology and Immunology, Amsterdam UMC, Location VUmc, 1081 HZ Amsterdam, The Netherlands; s.gibbs@amsterdamumc.nl; 3 Amsterdam Institute for Immunology and Infectious Diseases (AII), Amsterdam UMC, 1081 HZ Amsterdam, The Netherlands; 4 Amsterdam Movement Sciences (AMS) Institute, Amsterdam UMC, 1081 HZ Amsterdam, The Netherlands; 5 Department of Oral Cell Biology, Academic Centre for Dentistry Amsterdam (ACTA), University of Amsterdam and Vrije Universiteit Amsterdam, 1081 LA Amsterdam, The Netherlands; 6 Alliance of Dutch Burn Care, 1940 EB Beverwijk, The Netherlands; 7 Burn Center and Department of Plastic and Reconstructive Surgery, Red Cross Hospital, 1940 EB Beverwijk, The Netherlands; 8 Paediatric Surgical Centre, Emma Children’s Hospital, Amsterdam UMC, Location AMC, 1105 AZ Amsterdam, The Netherlands

**Keywords:** functional hydrogels, burn treatment, wound healing, skin regeneration, tissue engineering, biomaterials

## Abstract

Burn injuries are complex and require effective wound management strategies. Traditional treatments, such as dermal templates, are limited by simplified extracellular matrix (ECM) composition (e.g., collagen-elastin or collagen-glycosaminoglycan), sheet-based formats, and frequent use of animal-derived materials. These limitations can reduce wound conformity, biocompatibility, and integration with host tissue. Functional hydrogels are being explored as alternatives due to properties such as high water content, biodegradability, adhesiveness, antimicrobial activity, and support for angiogenesis. Unlike standard templates, hydrogels can adapt to irregular wound shapes as in burn wounds and reach deeper tissue layers, supporting moisture retention, cell migration, and controlled drug delivery. These features may improve the wound environment and support healing in burns of varying severity. This review outlines recent developments in functional hydrogel technologies and compares them to current clinical treatments for burn care. Emphasis is placed on the structural and biological features that influence performance, including material composition, bioactivity, and integration capacity. Through an exploration of key mechanisms of action and clinical applications, this review highlights the benefits and challenges associated with hydrogel technology, providing insights into its future role in burn care.

## 1. Introduction

Burn injuries represent a significant global health challenge, with both physical and psychosocial consequences. According to the World Health Organization (WHO), approximately 11 million burn injuries occur worldwide each year, leading to around 180,000 fatalities. Non-fatal burns often result in prolonged hospitalizations, long-term disabilities, and significant social prejudice, all of which can severely affect a patient’s quality of life. The impact on patient quality of life as well as the socioeconomic burden of burn care highlight the urgent need for continued innovation in burn treatment and care strategies. The development of advanced treatment options, such as hydrogel-based dressings, presents a promising solution for managing burn injuries of varying degrees and types, as hydrogels enhance wound healing, provide pain alleviation, and help prevent infections [1,2].

**Figure 1:**
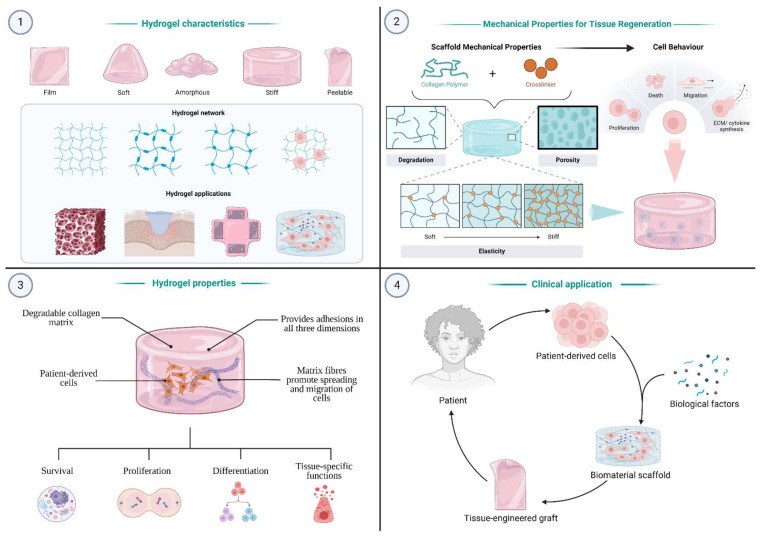
Overview of hydrogel-based tissue engineering. (**1**) Hydrogels exhibit diverse physical forms and internal networks, enabling various biomedical applications. (**2**) Mechanical properties (e.g., stiffness, porosity) can be tuned via polymer crosslinking to influence cell behavior and tissue regeneration. (**3**) Hydrogels support 3D cell adhesion, migration, and matrix remodeling, promoting survival, proliferation, differentiation, and tissue-specific functions. (**4**) Patient-derived cells and biological factors are combined with hydrogel scaffolds to create tissue-engineered grafts for clinical implantation. (Created with Biorender.com).

A hydrogel is defined as a hydrophilic polymer with a wide range of applications in health, research, and industry (Figure 1). It is a three-dimensional network structure consisting of a water-soluble polymer and a liquid with a cross-linking agent, allowing it to hold large volumes of water [3]. Its composition mimics that of the natural extracellular matrix (ECM), making it an invaluable candidate for tissue engineering. Moreover, hydrogel is promising in the context of wound management due to its biodegradability, adhesivity, anti-inflammatory, pre-angiogenic, and antimicrobial bioactivities, all of which contribute to an accelerated healing process [4,5,6].

**Figure 2:**
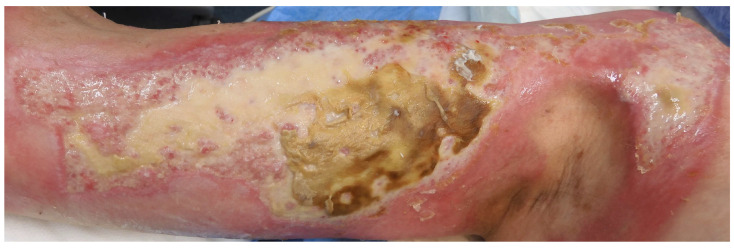
Photograph of a burn wound showing irregular wound edges and varying depths. The wound’s uneven contours highlight the potential benefit of hydrogels, which can conform to irregular wound shapes, penetrate deeper tissue layers, and support critical healing processes such as moisture retention, cell migration, and potentially facilitate, e.g., controlled drug delivery.

Hydrogel dressings have emerged as innovative solutions that can act as space-filling agents and vehicles for the delivery of therapeutic molecules, accelerating tissue repair. Unlike standard templates, hydrogels can adapt to irregular wound shapes as in burn wounds (Figure 2) and reach deeper tissue layers, supporting moisture retention, cell migration, and controlled drug delivery. The capacity for modulation in hydrogels allows for the development of a variety of functional dressings, making it one of their most promising features. Functional dressings can be made by incorporating various organic and inorganic materials, from antimicrobial metal oxides to anti-inflammatory hyaluronic acid, to electrically conductive polyaniline. This functionality allows hydrogel dressings to be modulated to suit the condition of burns caused by electrical, chemical, radiation, and thermal injuries [7].

The main objective of this narrative review is to give an overview of the current state of knowledge regarding the potential of functional hydrogels in burn treatment. By examining the key mechanisms of action and clinical applications, this review underscores the benefits and challenges of hydrogel technology, offering insights into its potential future role in burn care.

## 2. Burn Wound Management: Characteristics and Therapeutic Interventions

### 2.1. Burn Wound Categorization

To provide context for the potential of functional hydrogels in burn treatment, it is essential to first understand the categorization of burn wounds. Burns are most commonly caused by thermal injuries, but can also be caused by electricity, chemicals, or radioactive materials. The severity of the body’s response depends on several factors, primarily the depth and surface area affected, which result in different combinations of symptoms, including pain, redness, swelling, blistering, and loss of sensation in the skin. As the severity increases, so does a patient’s morbidity and mortality. Therefore, it is imperative to classify a burn accurately to guide management and determine outcomes [1].

Burns are primarily classified by their depth. A superficial partial-thickness burn affects the epidermis as well as the superficial layer of the dermis, and heals without intervention, with minimal scarring. Deep partial-thickness burns exhibit similar features, but also affect the deeper reticular dermis, resulting in a larger scar. Full-thickness burns involve the epidermis, dermis, as well as the subcutaneous layer. Such wounds result in a stiff, dry, and leathery appearance and do not blanch with pressure due to compromised blood supply. They require surgical intervention and take more than 8 weeks to heal. Considering the clinical implications of the burn categories described above, the following sections will focus on burns affecting the dermis layer, namely, deep partial-thickness and full-thickness burns, as these present greater challenges in healing and management. The following sections therefore address the post-burn immune response, common complications, and current therapeutic interventions relevant to these clinically significant burn types [1,8,9].

### 2.2. The Post-Burn Immune Response

Understanding the body’s complex immune response to severe burns is crucial for appreciating the potential of hydrogel technology in burn care. This response occurs both locally at the wound site and systemically, involving three overlapping stages of wound healing: inflammation, proliferation, and remodeling, ultimately leading to scar formation. By examining these stages and the associated pathologies, we can better grasp the benefits and challenges of hydrogel technology in burn treatment [7,8,10,11].

In most non-burn injuries, the body’s initial local response to tissue damage is hemostasis, involving arterial contraction to limit blood loss, platelet plug formation, activation of the coagulation cascade, and the formation of a clot or fibrin plug. This is followed by inflammation, during which exudates and immune cells are transported to the wound site via vasodilated blood vessels. In contrast to the typical healing process observed in non-burn injuries, burn injuries do not undergo a standard hemostasis phase. This deviation is primarily due to the thermal damage that destroys blood vessels. While non-burn injuries involve vessel constriction and clot formation to limit blood loss, burn wounds are distinctively characterized by immediate tissue necrosis and capillary damage. This results in fluid loss and inflammation, bypassing the typical hemostatic process seen in healthy healing. Additionally, various growth factors, pro-inflammatory cytokines, toxic proteases, and reactive oxygen species (ROS) are released into the wound tissue, eliminating invading microorganisms and removing necrotic tissue from the wound bed. The subsequent stage of proliferation in burn wound healing involves the formation of an ECM and granulation tissue, which consists of new blood vessels and connective tissue. However, unlike normal wound healing, this process in burn wounds often leads to significant scarring. The final stage, remodeling, is characterized by the regression of blood vessels and the reconstruction of the ECM. In burn wounds, the ECM does not typically reconstitute to resemble healthy tissue. Instead, due to extensive damage and the body’s response, the ECM often results in fibrous scar tissue formation. This scar tissue lacks the elasticity and tensile strength of healthy skin, leading to functional and aesthetic impairments [1,7,10,11,12,13,14].

By exploring these key mechanisms of action and clinical applications, the potential of hydrogel technology in burn care becomes evident, highlighting its benefits and challenges and offering insights into its future role.

### 2.3. Interventions and Treatments

Complications within the burn wound healing sequence can lead to severe consequences, resulting in adverse health outcomes. The main complications include bacterial infections, secondary infections and sepsis, pathological wound deepening, poor scarring, edema, and nerve damage. These complications underscore the need for interventions to protect and promote the healing and rehabilitation processes, highlighting the potential benefits of hydrogel technology in managing these challenges [3,11,14,15].

#### 2.3.1. Skin Grafting

The most common treatment for partial and full-thickness burn wounds involves early excision and grafting. Early excision attenuates the hypermetabolic state and reduces the risk of sepsis by removing debris and necrotic tissue, which can otherwise become a breeding ground for infection. Grafting further supports the healing process by closing the wound, which helps prevent infection, enables earlier movement, and decreases pain. It also reduces granulated tissue formation, thereby improving scar quality. The aim of this surgical approach is to remove all burned tissue, debride the area down to viable tissue, and close the wound [15].

In stable patients with viable donor sites post-resuscitation, the standard procedure is an autologous split-thickness skin graft. Skin from an uninjured donor site is removed using a dermatome and placed on the excised burn wound site. Meshing techniques are often employed to address insufficient donor skin coverage. Meshing involves making small incisions in the donor skin, allowing it to stretch and cover a larger area. This technique reduces the need for extensive donor sites, although it may result in less aesthetically pleasing scars due to the visible mesh pattern.

The Meek technology offers an advanced solution by allowing donor skin to be meshed at a higher ratio, such as 1:9. This technique involves cutting the skin into small squares and stretching them to cover a larger area. The uncovered wound areas can then be treated using a dermal regenerative matrix and/or a cell suspension spray, which helps alleviate some of the associated morbidity [16]. In cases where autologous skin grafting is not feasible due to the extent of the total body surface area (TBSA) burned, temporary coverage is required to minimize the complications of open excisions. Alternative skin substitutes include porcine skin (xenograft) or cadaver skin (allograft), which may provide coverage for up to 14 days before inevitable rejection [17,18].

##### Limitations and Challenges of Skin Grafts

The failure of skin graft engraftment can occur due to various complications, including inadequate excision of the wound site, the formation of a barrier between the wound bed and the graft, and infection at the wound site [18,19]. The success of a skin graft is highly dependent on the metabolic stability of the recipient site, which is influenced by nutritional support. Initially, the graft relies on the diffusion of nutrients from the wound bed, followed by the process of neovascularization, where new blood vessels grow into the graft, ensuring a continuous supply of nutrients and oxygen. Disruption of this nutrient and oxygen supply can occur due to several factors, primarily the separation of the graft from the wound bed. This separation can be caused by mechanical stress, infection, or the formation of a hematoma or seroma. Although graft failure is relatively uncommon in clinical practice, rejection rates can be as high as 33% in certain areas, such as lower-limb skin grafts [18,19].

Skin grafts, despite their relatively high general success rate, are not suitable for all patients and do not address all issues related to recovery from burn injuries. Patients who are unstable during resuscitation, have active bleeding or infections, or have a high percentage of TBSA affected with insufficient donor sites are ineligible for skin grafts [9]. The critical 36-h resuscitation period and the immunocompromised state often present at admission can lead to a high risk of morbidity and mortality due to infections. Relative contraindications for autologous skin grafts include malnourishment, smoking, bleeding disorders, use of anticoagulants, and chronic corticosteroid use [2,20]. Additionally, advanced age is a significant factor for worse outcomes in morbidity and mortality, as well as longer rehabilitation times and more pronounced post-operative complications [21].

Furthermore, skin grafting alone does not address several issues affecting immediate and long-term recovery from burn injuries. Infection, re-epithelialization, and nerve damage are primary concerns that are not directly addressed by skin grafts without additional interventions [22]. Skin grafts primarily serve as a barrier to protect against infection, and antimicrobial ointments and antibiotics are often still necessary to combat the high prevalence of infection. Re-epithelialization, which is critical for healing, is directed by stem cells residing in hair follicles in the dermis. Improper donor site excision or thin split-thickness skin grafts may lack the necessary cells for proper re-epithelialization [23,24]. In full-thickness burns, where nerves and blood supply in the subdermal plane are destroyed, patients may lose sensation or experience chronic pain. Although neuropathy is a significant concern, it remains an area requiring further research and innovation. With a prevalence of up to 52%, issues like chronic pain and sensory loss highlight the need for continued advancements in burn care [25,26,27].

Recognizing these limitations emphasizes the importance of advanced technologies in burn care. By delving into key mechanisms and clinical applications, we can underscore the potential benefits and challenges of innovative solutions like hydrogel technology, thereby offering valuable insights into the future of burn treatment.

#### 2.3.2. Artificial Substitutes

The limitations of conventional skin grafts can be addressed through various methods, primarily tissue-engineered skin consisting of cells and/or an ECM. The composition, sourcing, functionality, and application of these substitutes vary widely. Skin substitutes can be categorized based on several factors, including composition (cellular or acellular), layers (single or double-layered), structure (epidermal or full-thickness dermal), the type of biomaterial they are made from (biological, biosynthetic, or synthetic), and the duration of cover (temporary, semi-permanent, or permanent) [28]. Dermal substitutes are defined as biomatrices that mimic the structure and function of the natural ECM and dermis, aiming to enhance proper wound healing and minimize scarring [25,29].

##### Acellular Dermal Matrices

Among the most popular substitutes for conventional skin grafting are acellular dermal matrices (ADMs), with AlloDerm being one of the most studied and used. AlloDerm serves as a relatively successful replacement for the dermis layer. Sourced from cadaver tissue, the epithelial layer is removed in a salt bath and the tissue is de-epithelialized via freeze-drying while maintaining the basal membrane. It is then used in conjunction with a split-thickness skin graft (STSG), providing an adequate bed for its proper adhesion [30].

Another widely implemented ADM is Glyaderm, the first non-profit dermal substitute consisting of collagen and elastin fibers derived from glycerol-preserved human allogenic skin. Glyaderm is indicated for full-thickness burns as well as bilayer skin reconstruction. A study evaluating Glyaderm in a randomized control trial compared the scar formation of STSG to STSG + Glyaderm. A year after grafting, it was found that STSG + Glyaderm scars were significantly more elastic than those of STSG alone [31].

Matriderm, composed of type I (major component) and type III (minor component) bovine collagen and elastin hydrolysate, is a single-layer dermal substitute. A study evaluating Matriderm application in conjunction with STSG histologically investigated its integration into the skin graft. Serial biopsies several weeks and months after the procedure evaluated the wounds and found evidence of early inflammatory infiltrate and vascularization as early as two weeks post-procedure. Evaluation of the biopsies also found resorption of the ADM at three weeks, demonstrating that Matriderm resorbs earlier than other available ADMs [32].

Integra, another widely available ADM, uses a silicone layer to protect the wound from infection and fluid loss during the initial stages of neodermis reconstruction and vascularization. When integration into the wound bed is complete, the silicone layer is replaced with an autologous STSG in the second part of a two-stage procedure. The template itself is composed of a bilaminate sheet of shark glycosaminoglycans with cross-linked bovine tendon collagen, as well as the silicone sheet cover. Integra has been widely studied in the context of scalp injuries and has been found to be more cost-effective than conventional alternatives in wounds exceeding 100 cm^2^. It was also found to be compatible with pre- and post-operative radiation, making it useful in the treatment of oncological patients [33].

These advancements in ADMs highlight their potential benefits and challenges, underscoring the need for ongoing research and innovation to address issues such as chronic pain and sensory loss, thereby providing valuable insights into the future of burn treatment.

##### Criteria for Burn Dressings

In recent years, the field of burn care has evolved to take a more comprehensive approach, considering not only survival but also the quality of life and functional outcomes. This shift means that therapeutic approaches must fulfill criteria beyond mere mortality prevention, addressing long-term complications such as infection, hypertrophic scar formation, nerve damage, hair growth, temperature regulation (sweat glands), and chronic pain at the site of the initial injury. The rapid advancements in tissue engineering have significantly improved the quality of outcomes by managing these nuanced issues associated with burn injuries [25,29].

To address these issues, burn dressings should possess several key properties. They must be biocompatible, biodegradable, have a porous structure, and appropriate mechanical properties. Additionally, they should provide a barrier against bacterial invasion, have an appropriate rate of water-vapor transmission to prevent wound immersion, and exhibit adequate adhesion to the wound surface while remaining accessible for removal [34]. Hydrogels meet these criteria more effectively than most dermal substitutes. Their unique characteristic lies in their capacity to be modulated through the incorporation of cells or various substances that can enhance healing [17,25,29].

Hydrogel technology, in particular, shows promise in addressing many of these challenges. By offering a versatile platform that can be tailored to specific therapeutic needs, hydrogels provide valuable insights into the future of burn treatment. However, addressing chronic pain and sensory loss remains a significant challenge, underscoring the need for ongoing research and innovation in this field.

## 3. Comprehensive Applications of Hydrogels in Burn Care

### 3.1. Hydrogel Structure, Properties and Advantages for Burn Treatment

Hydrogels are water-rich polymers whose characteristics are determined by the type of polymer and the nature of their cross-linking. They can be formed using various cross-linking methods, with the main approaches being physical and chemical cross-linking. Physical cross-linking involves intermolecular interactions such as hydrogen bonding, crystallization, and hydrophobic interactions. The main advantages of physically cross-linked hydrogels are their high biocompatibility and low molecular toxicity. Chemical cross-linking refers to intramolecular interactions, including covalent bonding, utilizing radiation and free radical polymerization. These hydrogels, therefore, have a broader range of mechanical properties. Additionally, polymers can be of natural or synthetic origin. Natural polymers are primarily polysaccharides, such as cellulose, dextran, chitosan, pectin, carrageenan, starch, alginates, pullulan, and chitin. Naturally-derived hydrogels, which are generally physically cross-linked, are commonly used in various biomedical applications due to their high biocompatibility [31]. Synthetic hydrogels, on the other hand, generally have a higher molecular weight and lower biocompatibility but a higher capacity for modulation and precise control of physical and chemical properties [25,32].

#### Advantages of Hydrogels

Hydrogels possess significant advantages in the treatment of burn wounds. Hydrogels can absorb an amount of water equivalent to thousands of times their dry weight, making way for their liquid-like diffusion capacity which allows them to absorb wound exudate. This characteristic also allows them to maintain an optimal moist environment for the healing process [35,36]. Their ability to maintain a moist wound environment promotes cell migration and proliferation, which are essential for rapid tissue regeneration. By preventing excessive dryness, hydrogels reduce cellular stress and encourage the growth of new epithelial cells, leading to faster wound closure. Additionally, hydrogels limit fibrosis and excessive collagen deposition, both of which contribute to hypertrophic scarring. By creating an optimal healing environment, they help produce smoother, more elastic skin, reducing long-term scarring and improving cosmetic outcomes. One of the key advantages of hydrogels is their adaptability to various wound types and locations. Available in sheet, gel, and spray formulations, they can conform to irregular wound surfaces, making them particularly useful for challenging areas such as joints, fingers, and facial burns. Hydrogels are also suitable for different burn severities, including partial-thickness burns, donor sites, and chronic wounds.

Hydrogels mimic the structure and function of soft tissue through their physical properties. They have mechanical properties similar to solids but the diffusion capacity of liquids, meaning they can release and absorb water in response to environmental stimuli and physiological variables. Research into hydrogels has focused on “smart” hydrogels with the capacity to respond to these stimuli, reflecting their ability to undergo rapid volume phase changes and structural changes in response to the stimuli [37,38]. These reactions can be triggered by changes in the environment, with temperature and pH being the most studied. Recently, there have been developments in hydrogels capable of responding to multiple stimuli simultaneously [39]. This capacity shapes the implications of hydrogels in burn treatment [25,32]. These stimuli-responsive behaviors allow smart hydrogels to dynamically adjust their swelling, degradation, and drug release profiles in response to the evolving burn wound microenvironment. For example, pH-responsive hydrogels can selectively release antimicrobial agents in acidic, infected wounds, where the drop in pH acts as a trigger [40]. Thermo-responsive hydrogels can modulate their structural integrity or release kinetics based on local temperature increases caused by inflammation [41]. Light- or enzyme-responsive systems offer even more precise control in advanced care settings. The development of multi-stimuli-responsive hydrogels, which integrate multiple physiological signals such as pH and temperature, enhances the ability to tailor hydrogel behavior to complex wound dynamics. These capabilities are especially valuable in burn treatment, where wound characteristics change rapidly over time and require adaptable therapeutic strategies.

**Figure 3:**
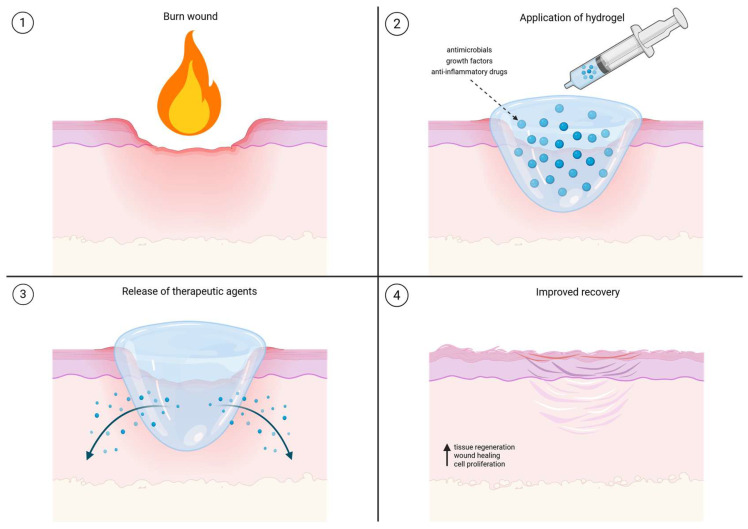
Hydrogel-based therapy for burn wound healing. (**1**) Burn injury induces skin tissue damage and inflammation. (**2**) Hydrogel loaded with therapeutic agents is applied to the wound site to provide a moist environment and deliver bioactive molecules. (**3**) Controlled release of therapeutic agents from the hydrogel promotes anti-inflammatory effects and cellular recruitment. (**4**) Enhanced tissue regeneration, wound healing, and cell proliferation, resulting in improved skin repair. (Created with BioRender.com).

The advantages of hydrogels can be further improved by expanding their functionality via the incorporation of drugs and therapeutic agents (Figure 3). Hydrogels that release drugs and therapeutic agents may provide a controlled and sustained release of agents in response to environmental stimuli [7]. Their capacity to be modulated via the implementation of cells and various chemicals means they can be customized further to suit the needs of the wound. The impregnation of dressings with, for example, antimicrobial agents, pain relievers, or growth factors enhances their therapeutic potential, making them a multifunctional option in burn care. Recent studies have also demonstrated that bioactive hydrogels can actively support tissue regeneration through additional biological mechanisms. For instance, hydrogels incorporating vascular endothelial growth factor (VEGF) have been shown to enhance angiogenesis by promoting endothelial cell proliferation and new blood vessel formation, an essential process for supplying nutrients and oxygen to healing tissue. The hydrated, porous matrix of hydrogels mimics the natural extracellular environment, supporting cell adhesion and directed migration, particularly for endothelial and epithelial cells. Moreover, some hydrogel formulations have been observed to influence the immune response by encouraging M2 macrophage polarization, which is associated with anti-inflammatory signaling and tissue repair. In parallel, specific hydrogel properties such as stiffness and degradability can affect fibroblast behavior and reduce fibrosis by downregulating pro-fibrotic factors like transforming growth factor-beta (TGF-β). These combined effects may help limit excessive scarring and improve overall wound healing outcomes [37,38]. Whether used alone or as part of a combination therapy, their versatility ensures customized treatment approaches for improved patient outcomes. Multiple clinical studies indicate that hydrogel dressings can significantly improve patient-reported outcomes in the context of burn wounds. For example, pediatric burn patients treated with Burnaid hydrogels reported lower pain scores and reduced use of pain-killers compared to traditional dressings. Patients with second-degree burns reported similar outcomes in that they experienced less pain during dressing changes and greater comfort with hydrogel sheets. In terms of scarring, hydrogel dressings for facial burns led to lower patient reported Vancouver Scar Scale scores and decreased hypertrophic scarring. While further research and clinical validation are ongoing, these findings highlight the potential of functionalized hydrogels to accelerate burn wound healing and improve scar quality through both physical and biological pathways [42,43,44].

### 3.2. Multifunctional Application of Hydrogels in Burn Care

#### 3.2.1. Surgical Applications of Hydrogels and Role as a Skin Substitutes

Hydrogels have emerged as a pivotal innovation in surgical applications, particularly in the realm of skin substitution and wound management. Their unique properties, such as high water content, biocompatibility, and the ability to mimic the ECM, make them ideal candidates for use as skin substitutes in surgical procedures. These characteristics facilitate a conducive environment for cell proliferation and tissue regeneration, which are crucial for effective wound healing and skin grafting [45,46].

In surgical settings, hydrogels are often utilized as temporary skin substitutes to cover and protect burn wounds and other extensive skin injuries. This application is particularly beneficial in cases where autografting is not immediately feasible due to the extent of the injury or the patient’s condition. Hydrogels serve as a protective barrier that maintains a moist environment, thereby reducing the risk of infection and promoting faster healing [47]. Additionally, their flexibility and adaptability allow them to conform to various wound shapes and sizes, making them suitable for complex wound geometries often encountered in surgical practice (Figure 2).

One of the significant advantages of using hydrogels as skin substitutes is their ability to integrate with host tissues. This integration is facilitated by the hydrogel’s porous structure, which allows for the exchange of nutrients and waste products, thereby supporting cellular activities essential for tissue repair [48]. Furthermore, hydrogels can be engineered to release therapeutic agents such as antimicrobials, growth factors, and anti-inflammatory drugs, which enhance their efficacy in promoting wound healing and reducing complications [49].

Recent advancements in hydrogel technology have led to the development of bioactive hydrogels that can actively participate in the healing process. These hydrogels are designed to interact with biological tissues at a molecular level, promoting cellular adhesion, migration, and differentiation. Such interactions are critical for the formation of new tissue and the restoration of skin function [50]. The use of bioactive hydrogels as skin substitutes represents a significant step forward in surgical wound care, offering the potential for improved clinical outcomes and enhanced patient recovery.

In conclusion, the role of hydrogels as skin substitutes in surgical applications is well-established and continues to evolve with ongoing research and technological advancements. Their ability to provide a supportive environment for tissue regeneration, coupled with their versatility and biocompatibility, underscores their importance in modern surgical practice.

#### 3.2.2. Antimicrobial Hydrogels

One of the major complications post-burn is bacterial infection at the wound site, particularly in the first week of hospitalization. The wound environment is especially susceptible to microorganism colonization, making hospital-admitted patients vulnerable to hospital-associated infections (HAIs) [51]. If a patient survives 72 h post-burn, infections become the most common cause of mortality. Multiple studies have shown that 42–65% of burn patient deaths can be attributed to infection [52,53,54,55].

Early resection and transplantation have reduced infection-associated mortality in burn patients. However, bacterial resistance is common, and it may be difficult for systemic antibiotics to reach local wounds; therefore, local delivery systems are of utmost importance. What is crucial for the prevention of infection is not only the delivery of antimicrobials but also isolating the wound from the environment, which may limit access to pollutants and bacteria [56]. Furthermore, controlling the environment to make it less favorable for pathogenic bacteria is also vital. Antimicrobial hydrogels have the capacity to absorb wound exudate while maintaining a sufficiently moist environment [57]. They also have the capacity to cover the wound, deliver antibiotics to treat infection in a precise, localized manner, and prevent bacterial colonization via the incorporation of antimicrobial materials [14].

While hydrogels do not inherently possess antimicrobial activity, their unique structure serves as a physical barrier that limits bacterial access to the wound and supports a balanced moist environment by allowing water evaporation and oxygen penetration. Importantly, hydrogels function as effective delivery platforms for antimicrobial agents, enabling localized and sustained infection control. In current practice, infection prevention may involve the application of betadine or chlorhexidine dressings over the hydrogel. Hydrogels can be made effective against infection through the incorporation of various antimicrobial compounds, for which the porous structure serves as an ideal vector. Wang et al. found that polysaccharide-based hydrogels may be cross-linked with essential oils, including terpenoids and terpenes, which have been found to be effective against Gram-positive and Gram-negative bacteria. Another natural agent that can be incorporated into the gel is honey, which has been shown to reduce the secretion of pro-inflammatory cytokines, including interleukin 1α, interleukin 1β, and interleukin 6 (IL-1α, IL-1β, and IL-6) [58,59].

Kim et al. also found antibacterial success with a thermo-sensitive methylcellulose injectable hydrogel containing silver oxide nanoparticles. Ionized silver nanoparticles have been found to create unfavorable conditions for bacterial growth and are implemented into multiple effective dressings. Dressings containing silver nanoparticles successfully inhibit pathogenic growth, including strains such as *Pseudomonas aeruginosa*, *Staphylococcus aureus*, including Methicillin-susceptible *Staphylococcus aureus* and Methicillin-resistant *Staphylococcus aureus* (MSSA and MRSA), and *Enterococcus faecalis* (VRE). They have also been found to delay the formation of biofilms on the wound surface. Silver nanoparticle-containing hydrogels are not cytotoxic, making for an effective and biocompatible burn wound dressing [60,61,62].

Novel solutions are required to combat common infection-causing bacteria such as MRSA and *Pseudomonas aeruginosa*, which pose a significant burden for burn patients [63]. Antimicrobial hydrogels may prevent the systemic effect of MRSA by sustaining local delivery of antibiotics [64]. Chibber et al. designed an anti-biofilm chitosan-based hydrogel aimed at the local administration of moxifloxacin [65]. Furthermore, Zhu et al. developed heme-rich Dex-HA hydrogels that effectively inhibited MRSA as well as *Escherichia coli.* Zhu et al. also developed a hydrogel that ensured the sustained release of colistin, conferring microbial resistance against *P. aeruginosa* [66]. Other antibiotic-containing hydrogels include an agarose-based gel developed by Grolman et al. The team found agarose-based gels with high concentrations of gentamicin or minocycline to maintain the stability of the antibiotics for over 7 days in porcine burn models [67]. The effectiveness of their gel was comparable to SSD cream, which is commonly used in practice, in the reduction of bacteria and burn depth [68].

The implementation of probiotics is a novel method of preventing and healing infections [69]. Oryan et al. found a type-I collagen-based hydrogel containing *Saccharomyces cerevisiae* antagonized pathogenic strains, including *Klebsiella pneumonia*, *P. aeruginosa*, *S. aureus*, and *Bacillus subtilis* [70,71,72]. The topical application of probiotics also had an immunomodulatory effect that decreased the severity of inflammation at the wound site [68].

#### 3.2.3. Hydrogels in Nerve Damage Treatment

Hydrogels present a promising approach to addressing various pathologies associated with peripheral nerve damage from burn injuries. By delving into how hydrogels work and their practical uses in medicine, we can better understand their advantages and limitations, shedding light on their promising future in treating burns. These pathologies include systemic abnormalities in nerve fiber density and sensory abnormalities, among others. Peripheral nerve damage can result in the denervation of target muscles, potentially leading to muscle atrophy [73]. These issues particularly affect patients with partial and full-thickness burns, where sensory axons are often damaged [74]. Unfortunately, skin grafting does not adequately address these issues and can sometimes exacerbate them [22]. Studies have shown increased levels of substance P-positive nerve fibers in grafts, which are associated with pain and itching [75]. Additionally, investigations into the loss of cutaneous sensibility post-burn have found that 97% of patients experience a significant decrease in response to various skin surface stimuli on the graft [76]. The conventional approach to treating peripheral nerve injury (PNI) involves autologous nerve transplants. However, this method has several downsides, including the need for multiple surgeries, potential further nerve damage, and pathological scar formation [77].

Tissue engineering strategies often employ natural and/or synthetic biomaterial-based nerve-guide conduits. These conduits bridge the stumps on either side of an injured nerve, providing structural and nutritional support and aiding in axon regeneration along the conduit. Traditional conduits, however, often lack flexibility and may not be fully compatible with nervous tissue, potentially causing local damage to the nerve stump [78,79].

Hydrogels are among the tissue-engineering materials that may serve to mimic certain aspects of the natural microenvironment of the nervous system, potentially aiding in the recovery of impaired neural function and the growth of new nervous tissue. Their three-dimensional cross-linked structure, capable of absorbing large amounts of water, provides a soft and elastic morphology that minimizes irritation to adjacent tissue in vivo. These inherent biological and physicochemical properties, along with a porous structure, tunable biodegradability, biocompatibility, and low immunogenicity, make hydrogels a promising candidate for neural tissue engineering [80].

Research into the implementation of hydrogels in the treatment of PNI includes investigating the following strategies: (1) utilization of factors that promote the differentiation of stem cells, (2) nerve growth factor use on the regeneration of peripheral nerves, (3) stimulating the regeneration of peripheral nerves according to their electrical conductivity [81]. Electrical signals can control the adhesion, proliferation, migration, metabolism, and differentiation of nerve cells. Although the mechanism is not fully understood, local changes to the electrical field and the release of neurotrophic factors, both important in the growth of nerves, are dependent on electrical stimulation [82]. Therefore, hydrogels with conductive properties may be the focal point of future research. Additionally, several studies have used the above-mentioned strategies individually and in combination with promising results in promoting nerve regeneration [83,84].

Conductive polymer (CP)-based conductive hydrogels, such as those derived from polyalanine, polypyrrole, and polythiophene, are widely used in electrically sensitive tissues such as the heart, skin, skeletal muscle, and nerves. CP-based conductive hydrogels have been found to enhance the adhesion and proliferation of rat adrenal chromaffin cells, L929 fibroblasts, mesenchymal stem cells, and cardiomyocytes [79].

Another prospect of hydrogels relating to the nervous system is pain management by prolonging the action of local anesthetics [85]. Other than the acute pain following the initial insult and hospitalization, studies have found that some patients report chronic neuropathic pain for up to 5 years post-injury [86]. Such complex pain may require sustained localized delivery of anesthetics, for which hydrogels may be suited due to their minimally invasive application, with many injectable hydrogel systems available [87].

Expanding on the above mentioned prospects, several studies explored advanced hydrogel applications in the context of skin-associated nerve damage, including burn-induced peripheral nerve injury. This includes hydrogels loaded with mesenchymal stem cell (MSC)-derived exosomes, which have shown anti-inflammatory effects and support for axonal repair in preclinical models. Hydrogels containing neurotrophic factors such as NGF, BDNF, and GDNF have also been used to promote Schwann cell migration and guide nerve regrowth. In addition, conductive hydrogels made with materials like polypyrrole or graphene oxide have been studied for enhancing nerve signaling and functional recovery. Some systems also incorporate aligned nanofibers to mimic the native nerve structure and support directed regeneration. These developments suggest that hydrogels may contribute not only to pain relief but also to nerve repair in burn injuries [88,89,90,91].

#### 3.2.4. Hydrogel-Based Cooling and First-Aid Applications

Beyond their regenerative potential, hydrogels offer significant advantages in the acute management of burn injuries, particularly as first-aid cooling agents. Following a thermal insult, tissue damage evolves over time, with residual heat penetrating deeper skin layers during the first 15 min [92]. As water-rich tissue conducts heat efficiently, rapid cooling is critical to prevent burn progression. Experimental models have shown that cooling at 16 °C for 20 min within the first hour yields optimal outcomes [93]. While tap water is commonly recommended, its availability is not guaranteed, especially in low-resource settings, mass casualty events, or when hypothermia is a concern. Hydrogels, composed of hydrophilic groups (–NH_2_, –COOH, –OH, –CONH_2_, –SO_3_H), offer an effective alternative by stabilizing wound temperature through high water content (up to 96%). These hydrogels have already seen operational use in ambulances and military settings, where they maintain skin temperatures around 20.5 °C at the surface and ~33 °C subdermally [44].

In pre-hospital care, hydrogels serve as both a cooling and protective dressing, reducing pain, fluid loss, and infection risk before hospital admission. In the UK, 39% of emergency medical services and nearly 80% of fire departments employ hydrogel dressings [94]. Moreover, they are frequently applied by non-professional first responders; over 50% of burn patients in one study received such treatment before clinical care [92]. Their safety in paediatric populations further broadens their clinical utility, with 13% of paediatric burns in Australia treated using hydrogel dressings [95].

A key advantage of hydrogel dressings is their ability to be gently and selectively removed, facilitating transition to definitive wound coverage. Recent innovations have yielded dissolvable hydrogels that minimize trauma to regenerating tissue. Konieczynska et al. engineered a lysine-based hydrogel removable via thiol–thioester exchange, while Huang et al. developed an injectable carboxymethyl chitosan–cellulose nanocrystal composite that dissolves upon contact with an amino acid solution [34,94,95,96]. These features position hydrogels as versatile tools not only in tissue regeneration but also in acute burn care, offering both immediate and adaptable solutions in diverse clinical settings.

#### 3.2.5. Future Perspectives on Functional Hydrogels in Burn Care

Advancements in hydrogel technology suggest promising directions for improving burn wound management. In particular, stimuli-responsive (“smart”) hydrogels, which react to changes in pH, temperature, or local biochemical signals, offer potential for more precise therapeutic delivery and wound adaptation. Future developments may also include integration with biosensors for non-invasive wound monitoring and tailored drug release, reducing the need for frequent dressing changes. Additionally, hydrogels incorporating spatially organized signaling molecules may enhance tissue regeneration by supporting nerve repair and neovascularization. These evolving features underscore the ongoing potential of functional hydrogels to address complex challenges in burn care.

## 4. Conclusions

Through their unique physicochemical properties and functional versatility, hydrogels represent a transformative platform in burn care, not merely as alternatives to traditional dressings, but as dynamic therapeutic tools capable of modulating the wound environment, delivering bioactive agents, and supporting tissue regeneration. Their ability to be tailored for specific surgical and pre-hospital needs underscores their central role in modern burn management strategies.

While the replication of native skin architecture, including vascular, neural, and appendageal components, remains an aspirational goal, current hydrogel systems provide a structurally supportive matrix that promotes cellular activity and wound closure. Progress in bioengineering and materials science continues to push the boundaries of what hydrogels can achieve, with ongoing efforts focused on improving tissue integration, reducing scarring, and enhancing patient outcomes.

By examining their mechanisms of action and expanding clinical applications, this review highlights both the opportunities and limitations of hydrogel technology, offering a forward-looking perspective on its evolving role in surgical practice and acute burn care.

Despite their promising therapeutic potential, multifunctional and bioactive hydrogels face significant regulatory and manufacturing hurdles that must be overcome before widespread clinical adoption. Addressing these challenges through standardized safety evaluations, scalable production methods, and clearer regulatory pathways will be crucial to translating advanced hydrogel technologies from bench to bedside.

While multiple clinical studies confirm the effectiveness of conventional hydrogel dressings in burn care, particularly for partial-thickness wounds, clinical validation of newer multifunctional or stimuli-responsive hydrogel systems remains limited. Current evidence stems primarily from preclinical models, with only a few pilot trials reported. Further high-quality, randomized clinical trials are urgently needed to assess safety, efficacy, and long-term outcomes of these advanced systems in diverse burn populations.

## Data Availability

This study is a narrative review and does not contain any original data. All information presented is derived from previously published studies, which are cited accordingly.
